# The Metabolic Reprogramming Induced by Sub-Optimal Nutritional and Light Inputs in Soilless Cultivated Green and Red Butterhead Lettuce

**DOI:** 10.3390/ijms21176381

**Published:** 2020-09-02

**Authors:** Begoña Miras-Moreno, Giandomenico Corrado, Leilei Zhang, Biancamaria Senizza, Laura Righetti, Renato Bruni, Christophe El-Nakhel, Maria Isabella Sifola, Antonio Pannico, Stefania De Pascale, Youssef Rouphael, Luigi Lucini

**Affiliations:** 1 Department for Sustainable Food Process, Università Cattolica del Sacro Cuore, 29122 Piacenza, Italy; mariabegona.mirasmoreno@unicatt.it (B.M.-M.); leilei.zhang@unicatt.it (L.Z.); biancamaria.senizza@unicatt.it (B.S.); luigi.lucini@unicatt.it (L.L.); 2 Department of Agricultural Sciences, University of Naples Federico II, 80055 Portici, Italy; christophe.elnakhel@unina.it (C.E.-N.); sifola@unina.it (M.I.S.); antonio.pannico@unina.it (A.P.); depascal@unina.it (S.D.P.); youssef.rouphael@unina.it (Y.R.); 3 Department of Food and Drug, University of Parma, 43124 Parma, Italy; laura.righetti@unipr.it (L.R.); renato.bruni@unipr.it (R.B.); 4 Research Centre for Nutrigenomics and Proteomics (PRONUTRIGEN), Università Cattolica del Sacro Cuore, 29122 Piacenza, Italy

**Keywords:** *Lactuca sativa*, macrocations, light intensity, metabolomics, sub-optimal response

## Abstract

Sub-optimal growing conditions have a major effect on plants; therefore, large efforts are devoted to maximizing the availability of agricultural inputs to crops. To increase the sustainable use of non-renewable inputs, attention is currently given to the study of plants under non-optimal conditions. In this work, we investigated the impact of sub-optimal macrocations availability and light intensity in two lettuce varieties that differ for the accumulation of secondary metabolites (i.e., ‘Red Salanova’ and ‘Green Salanova’). Photosynthesis-related measurements and untargeted metabolomics were used to identify responses and pathways involved in stress resilience. The pigmented (‘Red’) and the non-pigmented (‘Green Salanova’) lettuce exhibited distinctive responses to sub-optimal conditions. The cultivar specific metabolomic signatures comprised a broad modulation of metabolism, including secondary metabolites, phytohormones, and membrane lipids signaling cascade. Several stress-related metabolites were altered by either treatment, including polyamines (and other nitrogen-containing compounds), phenylpropanoids, and lipids. The metabolomics and physiological response to macrocations availability and light intensity also implies that the effects of low-input sustainable farming systems should be evaluated considering a range of cultivar-specific positive and disadvantageous metabolic effects in addition to yield and other socio-economic parameters.

## 1. Introduction

Lettuce (*Lactuca sativa* L.) is the most developed model system in the Asteraceae (formerly, Compositae) family [1] and is the reference species for engineering plant factories and, more generally, for biological studies on vegetables in closed soilless systems. Although typically cultivated and used in Mediterranean countries (e.g., Italy, Spain, and France) [2], this leafy vegetable is recently experiencing a significant diffusion in different economically developed countries, mainly USA and China [3,4]. The main reason is the rising appreciation for healthy, low-calorie raw foods along with an overall improvement in post-harvest management, which more effectively guarantees the quality attributes of this easily perishable leafy green [5,6]. Moreover, breeding has not only introduced resistance to biotic stress, but it has also allowed the exploitation of the various leaf textures, shapes, and colors present in the lettuce germplasm, enlarging the range of horticultural types and cultivars that are regularly available to consumers [7]. Another factor that contributes to the success of lettuce is the diffusion of hydroponics (i.e., any method of growing plants in a water-based, nutrient-rich solution) [8]. Hydroponics is well suited to lettuce because of its short growth cycle, high added value, and minimal (plant) wastes, due also to the cut-and-come-again harvest strategy [9]. Moreover, hydroponics can considerably increase the quality (e.g., crispness, cleanness, phytosanitary conditions) and the uniformity (e.g., size, appearance, color) of lettuce heads, especially in plant factories and indoor modules with artificial lighting [4].

The nutritional and the phytochemical features of lettuce vary considerably among cultivar-groups (e.g., butterhead, crisphead, oakleaf, lollo, etc.) [2,10,11]. In general, lettuce is rich in ascorbic acid, vitamins, and carotenoids [12,13] as well as other antioxidants related to the phenylpropanoid biosynthetic pathway, such as flavonoids [14]. In butterhead types, the highest concentration of phenolic compounds is present in outer leaves, consistent with a direct influence of light for the accumulation of various classes of secondary metabolites [14,15]. The phytochemical composition of lettuce, such as that of any other plant, is also dependent on nutrient availability [16,17]. For instance, the reduction of nutrients to a level that does not result in deficiency symptoms can promote physiological responses that eventually result in a higher concentration of health-promoting plant secondary metabolites [18,19]. While there is ample evidence of the effect of light intensity and nutrient availability in crop and food sciences [11,15,17,20,21,22], relatively less is available on the cumulative, wide-ranging characterization of the biochemical processes that underline lettuce response to these factors. This knowledge is essential to translate functions and dynamics of this crop into a mechanistic understanding of physiological responses, a knowledge ultimately necessary to increase yield, quality, and sustainability (e.g., a more efficient use of non-renewable resources such as light energy and chemical fertilizers) [23]. Additionally, this information can provide new breeding targets (e.g., metabolic biomarkers) for the development of stress tolerant or resilient varieties [24].

This work aimed to test whether the response to suboptimal growing conditions can have markedly different metabolic outcomes according to the secondary metabolism of the plant. To this goal, we exploited two lettuce cultivars that differ in leaf coloration, ‘Red Salanova’ and ‘Green Salanova’, which in normal growing conditions develop red-pigmented and green-pigmented leaves, respectively. To elucidate the impact of the reduction of the availability of macrocations or of light intensity in these two genotypes, we performed a physiological and metabolomics analysis of leaf tissues of plants growing either on three nutrient solutions with a progressive reduction of macrocations or under two light-intensities (i.e., optimal and sub-optimal).

Our work provided a comprehensive view of the variations in two lettuce butterhead cultivars with a different accumulation of secondary metabolites according to two abiotic factors that strongly influence plant physiology. The study of the metabolic alteration also allowed a broader understanding of shared or tailored molecular processes that underlie lettuce adaptation to light intensity and mineral availability.

## 2. Results

### 2.1. Leaf Gas Exchange Analysis

The results of the physiological analysis of green and red-pigmented butterhead lettuce in relation to macronutrient solution concentration or light intensity are presented in Table 1 and Table 2, respectively. In the first experiment, an interaction between the macrocation concentration in the nutrient solution and the genotype was not observed for all the physiological parameters. When averaged over nutrient solution concentration, the net CO_2_ assimilation rate (A_CO2_), stomatal resistance (r_s_), and intrinsic water use efficiency (WUE_i_), were higher in the ‘Red Salanova’, whereas a contrasting trend was observed for transpiration rate (E) (fold). Conversely, when averaged over cultivars, A_CO2_ values decreased linearly when nutrient solution electrical conductivity (EC) decreased from 1.5 to 0.5 dS m^−1^ (Table 1).

Contrarily to the macronutrient solution concentration experiment, most of the measured physiological parameters (except WUE_i_) displayed a significant interaction between cultivar and light intensity (Table 2). The A_CO2_ and the E were higher under optimal light conditions (420 µmoL m^−2^ s^−1^) with red pigmented lettuce exhibiting the highest overall values (Table 2). Particularly, the A_CO2_ reduction under sub-optimal light intensity was more pronounced in red (46%) compared to green butterhead lettuce (40%; Table 2). The main effect of the low light intensity treatment (e.g., averaged over cultivars) on the WUE_i_ was a significant reduction (21.1%; Table 2). A_CO2_ and E were also significantly lowered by the same light treatment.

### 2.2. Metabolomics

An untargeted metabolomics approach was employed to gain insights into the biochemical processes affected by the different nutrient solution concentrations and light intensities in the ‘Green’ and ‘Red Salanova’ lettuce. The analysis of plant methanolic extracts showed a broad diversity of secondary metabolites, including polyphenols, phenylpropanoids, terpenes, but also more polar compounds involved in the TCA cycle as well as polar lipids (i.e., fatty acid and glycolipids). Overall, more than 4000 putative metabolites were annotated using the comprehensive database PlantCyc 12.6. The annotated compounds and composite mass spectra (mass and abundance combinations), together with compounds identified by MS/MS, are listed in Tables S1 and S2.

The effect of nutrients concentrations and light intensities were processed independently, assuming that both factors may play a diverse role in modulating the phytochemical profiles, as previously highlighted for targeted quantification of anthocyanidins and carotenoids [25,26]. Furthermore, distinct multivariate models were built for the two cultivars, ‘Green’ and ‘Red Salanova’, the cultivar being the main factor responsible for the sample’s arrangement, as pointed out by the unsupervised hierarchical cluster analysis (HCA) clustering (Figure S1).

#### 2.2.1. Effect of the Nutrient Solution Strength on Metabolic Profiles

**Figure 1:**
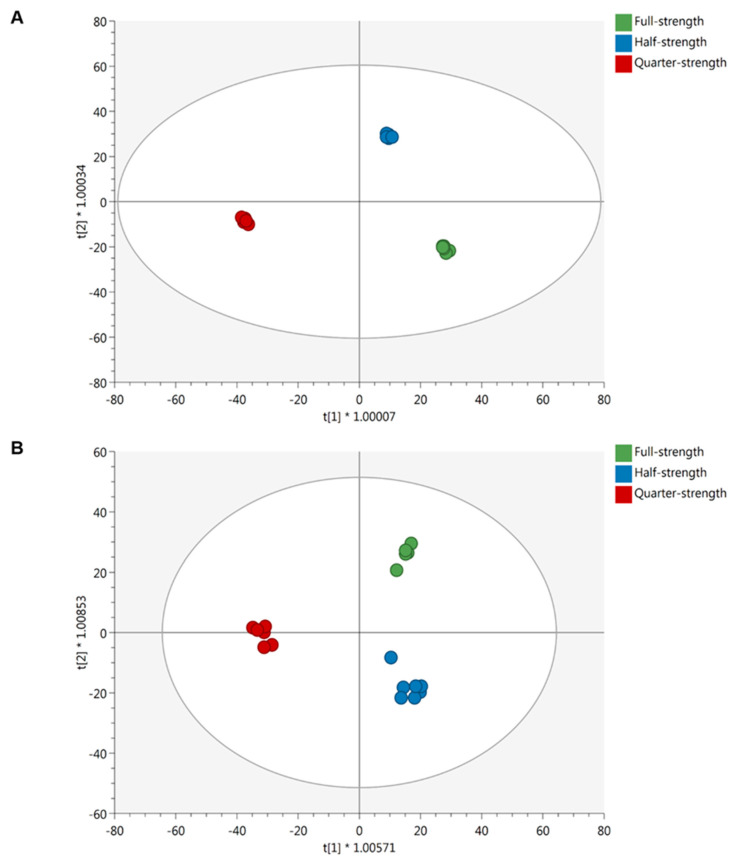
Score plot of orthogonal projection to latent structures discriminant analysis (OPLS-DA) supervised modeling carried out on untargeted metabolomics profiles of ‘Red’ (**A**) (R^2^Y = 0.99, Q^2^Y = 0.91) and ‘Green Salanova’ (**B**) (R^2^Y = 0.97, Q^2^Y = 0.69) subjected to different nutrient solutions.

As suggested by the unsupervised HCA (Figure S1), the plant metabolic profile is significantly influenced by the reduced concentration of mineral nutrients. The orthogonal projection to latent structures discriminant analysis (OPLS-DA) further confirmed the unsupervised samples clustering supervised modeling, which allowed us to separate the samples in the score space according to full-, half-, and quarter-strength nutrient solutions (Figure 1). Both ‘Red’ (Figure 1A) and ‘Green Salanova’ (Figure 1B) cultivars shared the same trend. The models were further validated by the goodness-of-fit (R^2^Y > 0.97), the prediction ability (Q^2^Y > 0.6), and by the cross-validated analysis of variance (CV-ANOVA) *p* values lower than 0.05. To elucidate the plant response to the reduction of nutrients, variable importance in projection (VIP) analysis was applied, resulting in 307 and 288 significant metabolites (VIP score > 1.3) for ‘Red’ and ‘Green Salanova’ comparisons, respectively. These metabolites were subjected to the fold-change analysis and then exported into the Pathway Tools Omics Dashboard for interpretation. The entire list of VIP markers is provided as Table S2 for ‘Red’ and Table S3 for ‘Green Salanova’.

**Figure 2:**
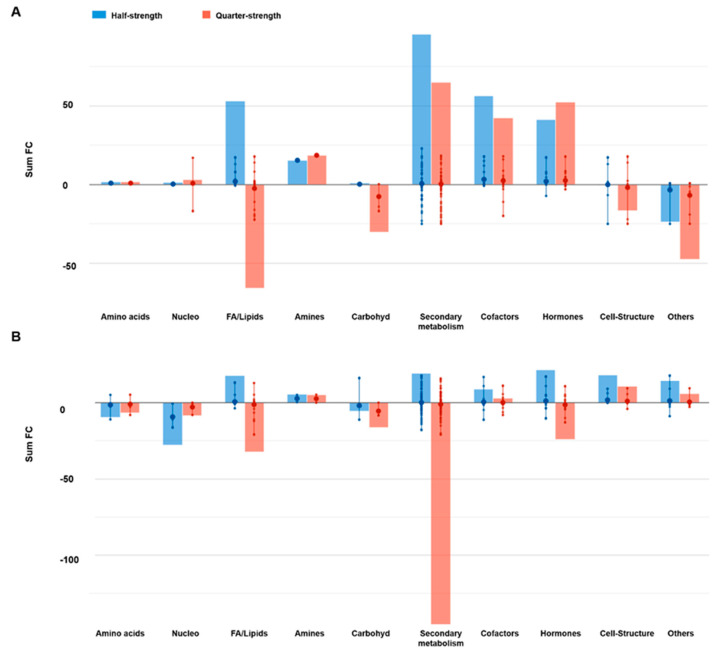
Processes affected by the macrocation concentration in ‘Red’ (**A**) and ‘Green Salanova’ (**B**). Differential metabolites and their fold-change (FC) values were elaborated using the Omic Viewer Dashboard of the PlantCyc pathway Tool software (www.pmn.plantcyc.com). In each class, the large dot represents the average (mean) logFC of the metabolites. Small dots represent the individual logFC for each metabolite. The abbreviated subcategory names on the *x*-axis correspond to: Nucleo: nucleosides and nucleotides; FA/Lipids: fatty acids and lipids; Amines: amines and polyamines; Carbohyd: carbohydrates; Cofactors: cofactors, prosthetic groups, electron carriers, and vitamins.

Overall, more than 300 compounds were involved in the response in both cultivars to macronutrient deprivation. Among these metabolites, only 57 compounds overlapped (Figure S2), indicating a cultivar-specific response to reduced nutrients availability. However, the macronutrient deprivation-mediated response was characterized by the reprogramming of secondary metabolism in both cultivars (Figure 2). A general accumulation of secondary metabolites was found for the red lettuce under reduced mineral availability. For ‘Red Salanova’, both half- and quarter-strength nutrient solutions elicited nitrogen-containing secondary metabolites and phenylpropanoids. A decrease of terpenes was found as a common response to reduced nutrition in all cases.

Besides secondary metabolism, cultivars also differed in the biosynthesis pattern of other molecules. Regarding the phytohormone profile, red lettuce presented a marked increase of auxins, cytokinins, and the gibberellin A_24_. In contrast, the opposite trend was observed for ‘Green Salanova’ with the decrease of cytokinins and the precursors of gibberellin. The abscisic acid (ABA)-related metabolites 7′-hydroxyabscisate and dihydroxyphaseic acid decreased in red and green lettuce, respectively, while the strigolactone carlactone increased in both cultivars. Several detoxifying and antioxidant molecules, such as glutathione or tocotrienol, were found in green rather than ‘Red Salanova’. Finally, porphyrin-related compounds were down-accumulated in the green-pigmented cultivar with reduced nutrition.

#### 2.2.2. Effect of Light Intensity on Metabolic Profiles

**Figure 3:**
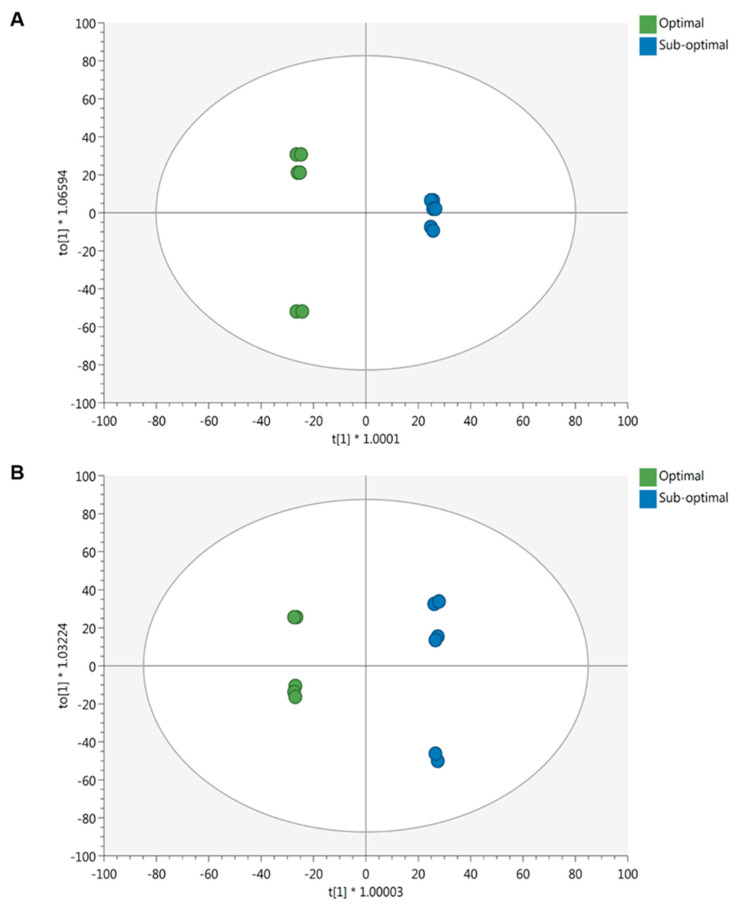
Score plot of OPLS-DA supervised modeling carried out on untargeted metabolomics profiles of ‘Red’ (**A**) (R^2^Y = 0.99, Q^2^Y = 0.92) and ‘Green’ (**B**) (R^2^Y = 1, Q^2^Y = 0.92) Salanova under optimal and sub-optimal light conditions.

Independent multivariate modeling was performed to specifically unravel the effect of light intensity on the phytochemical profile of lettuce. The whole dataset is provided as Table S4. The unsupervised HCA (Figure S3) revealed differences in metabolomic signatures as a function of the light intensity during growth conditions. Consistent results were achieved by the OPLS-DA (Figure 3) modeling. The lettuce metabolome revealed to be strongly influenced by the light intensity (optimal vs. sub-optimal), regardless of the cultivar considered.

As detailed for the macrocation analysis, the OPLS-DA model was validated, and significant metabolites were selected (VIP score > 1.3). Overall, 3908 metabolites were identified against the database PlantCyc, of which 358 and 330 revealed to be statistically significant for ‘Red’ and ‘Green Salanova’, respectively. These metabolites were subjected to pathways analysis for biological interpretation and are listed in Tables S5 and S6. The plant response appeared to be strongly influenced by the genotype, since only 99 out of 300 discriminant metabolites were shared between the two comparisons, accounting for 16% of the biologically interpreted metabolites (Figure S4).

**Figure 4:**
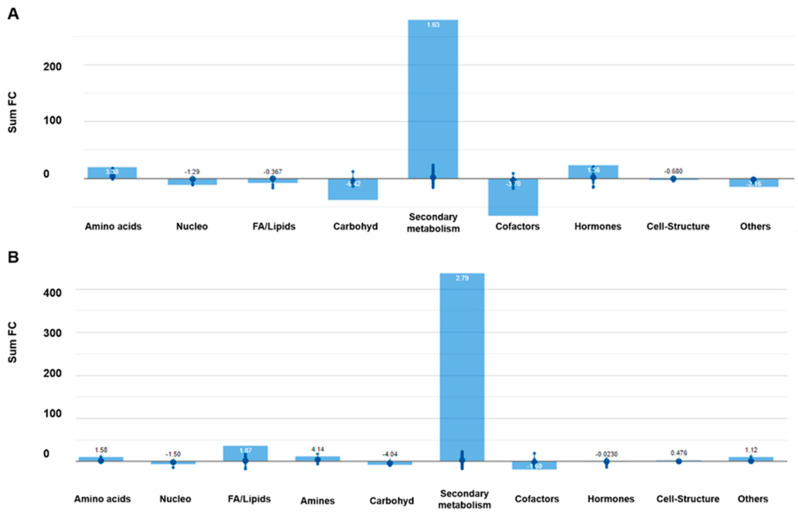
Processes affected by the light intensity in ‘Red’ (**A**) and ‘Green Salanova’ (**B**). Differential metabolites and their fold-change (FC) values were elaborated using the Omic Viewer Dashboard of the PlantCyc pathway Tool software (www.pmn.plantcyc.com). In each class, the large dot represents the average (mean) logFC of the metabolites. Small dots represent the individual logFC for each metabolite. The abbreviated subcategory names reported on the *x*-axis correspond to: Nucleo: nucleosides and nucleotides; FA/Lipids: fatty acids and lipids; Amines: amines and polyamines; Carbohyd: carbohydrates; Cofactors: cofactors, prosthetic groups, electron carriers and vitamins.

Irrespective of the cultivar considered, the largest impact was in secondary metabolism (Figure 4). Taking the high intensity as reference condition, the increase of secondary metabolites was positively correlated to the decrease of light intensity. Particularly, phenylpropanoids increased in both cultivars. However, several conjugated anthocyanins were down accumulated in ‘Green Salanova’, and other phenolic derivatives such as glyceollin or chrysoeriolin decreased in ‘Red Salanova’. Regarding nitrogen-containing secondary metabolites, glucosinolates were involved in the response to low-light intensity. Although a complex alteration was found, many compounds involved in the biosynthesis of glucosinolates increased in the green cultivar and decreased in the red cultivar. Terpenes were strongly elicited in the red cultivar and, to a lesser extent, in the green cultivar. Among these compounds, the tetraterpene phytoene strongly accumulated in both cultivars. A similar trend was found for different sterols, sesquiterpenes, and diterpenes. Similarly, the terpenic hormones brassinosteroids (BR) highly increased under the low-light condition. The diterpenic giberrellins A_24_, epoxy gibberellin A_4_ increased, while the gibberellin A_34_ and A_6_ decreased in the red cultivar. In contrast, its precursor *ent*-kaurenol and dihydro-dihydroxy gibberellin A_1_ accumulated in ‘Green Salanova’. Other phytohormones showed alterations after light condition modifications. Strigolactones were repressed in the green cultivar, while no changes were found in the red one. Cytokinins decreased in the green cultivar and, to a greater extent, in the red cultivar. Regarding jasmonates, its precursor oxo(pentenyl)-cyclopentane-(hydroxyoctanoyl)-CoA was slightly accumulated in the red cultivar, while jasmonoyl-l-phenylalanine and epi-oxojasmonoyl-l-isoleucine accumulated in the green cultivar. Finally, molecules related to reactive oxygen species (ROS) detoxification were found in a lower amount under low-light conditions. With this regard, cultivar-specific differences could be outlined. For instance, glutathione and its derivatives decreased in both cultivars, while, for instance, hexenone decreased in ‘Green Salanova’ and monodehydroascorbate radical in ‘Red Salanova’.

## 3. Discussion

The profile of plant metabolites is influenced by different abiotic factors such as light, temperature, water status, and nutrient availability [27,28,29]. Most of the studies investigating the effect of such factors on lettuce focused on phenolic compounds [26,30,31], a frequently used quality parameter for the evaluation of plants [32].

Thanks to untargeted metabolomics, in this work, we showed that macrocation availability and light intensity also affect pathways other than those leading to the production of polyphenols. Even though a strong effect of the abiotic factors has been corroborated, our work indicated that the metabolomics response of lettuce metabolome is strongly influenced by the different anthocyanin coloration. Moreover, photosynthetic performances such as carbon assimilation, transpiration, and water use efficiency were also different between the two cultivars, even under optimal conditions. Overall, the red-pigmented genotype was more responsive to the limiting nutrient solution compared to the non-pigmented (green) genotype. This is in line with a previous study reporting a significant increase of both phenols and carotenoids in the red compared to the green cultivar [30]. Here, we observed a strong activation of the secondary metabolism in the ‘Red Salanova’ under limiting macrocation availability, while a down-accumulation was observed in the ‘Green Salanova’. The largest differences were recorded for the nitrogen-containing metabolites and for the phenylpropanoid pathways. These were strongly triggered in the red-genotype and lowered in the green-genotype. Phenylpropanoids are key contributors to virtually all aspects of plant responses towards biotic and abiotic stimuli, and their biosynthesis is the result of the complex interactions between hormones and stress signaling [33]. Among other regulatory mechanisms, cytokinins can trigger the expression of stress-induced genes involved in plant secondary metabolism, such as flavonoid and phenylpropanoid biosynthesis [34]. Cytokinins are involved in plant development, but an important role in stress adaptation and response has been also established [35,36]. Here, we detected an accumulation of cytokinins, auxins, and abscisic acid in response to nutrient privation in ‘Red Salanova’, while the opposite trend was observed for the green genotype. The concurrent decrease of carbon dioxide assimilation was consistent with such hormonal imbalance and with the reduced porphyrin accumulation we observed. At the same time, photosynthetic performance evidenced a significant genotype–light interaction, with assimilation and WUEi decreasing at reduced light intensities.

The lipid fraction was also affected by the lowest nutrient concentration. We observed accumulation of phosphatidylcholines (PC), including PC (16:0/18:1) and PC (18:1/18:1) in ‘Red Salanova’. Phospholipids’ accumulation in stress conditions is associated with the activation of phospholipases (PL) and membrane modifications that occur during defense. Hong and colleagues demonstrated the involvement of PL in response to nitrogen starvation, as it is in the quarter-strength solution [37]. Nitrogen starvation also leads to galactolipids and chlorophylls breakdown [38,39]. A decrease of glycerolipids was observed in both genotypes, and porphyrin-related compounds were down-accumulated in the green-pigmented cultivar under reduced nutrition.

The impairment of polyamines and amino acids was also detected because of nutrient solution deprivation. Putrescine, spermidine, and spermine pathways were activated exclusively in the green genotype. These compounds play important roles, especially in relation to abiotic stresses, including salt, drought, extreme temperature, waterlogging, and toxic metals [29,40]. Arginine decarboxylase is responsible for the first step of polyamine biosynthesis with the production of the intermediate omithine; therefore, a reduction in arginine could be expected in ‘Green Salanova’. However, putrescine can also be synthetized indirectly from arginine through the proline pathways [41], which could also explain the decrease in Gln, His, Lys, Trp, and Phe detected in this study.

A common modulation of carbohydrate biosynthesis was observed in ‘Green’ and ‘Red Salanova’, together with a decrease of terpenes (the latter was a common response to reduced macrocation availability). The accumulation of terpenes allows plants to cope with abiotic and biotic stress. However, the influence of nutrient availability on terpenoid storage is complex [42]. For example, under nitrogen supply, terpenoid content in *Pinus sylvestris* needles was increased [22], unaltered [23], or impoverished in mature needles [24]. Other reports suggested an imbalance of terpenes under phosphorus starvation as a consequence of reduced amounts of phospholipids forming the bilayer in cell membranes [43]. In non-storing species, terpenoids are subjected to rapid responses to abiotic stress because of the absence of a terpenoid reservoir [42]. In the present study, terpenoid biosynthesis was reduced following nutrients’ reduction, while it increased in response to sub-optimal light intensity regardless of the lettuce genotype. This result is in line with Harley et al. [44], who reported the increase in terpenes in white oak leaf when applying two levels of light (300 and 800 µmoL/m^2^s).

Apart from terpenes, the biosynthesis of other secondary metabolites was triggered in response to sub-optimal light intensity. As observed under nutrient solution deprivation, also the intensity of light seems to stress the plant, which reacts by producing a higher amount of phenylpropanoid metabolites. The increase was particularly significant for the green genotype. On the other hand, the biosynthesis of fatty acids derivates was exclusively triggered in ‘Red Salanova’. Several oxylipins were found accumulated in response to light stress. Oxylipins are key signaling compounds that play essential roles in the adaptation to photo-oxidative stress by regulating the biosynthesis of the plant signaling molecules [45]. Accordingly, the biosynthesis of jasmonic acids (JA) was triggered solely in ‘Red Salanova’. The same trend was observed for brassinosteroids that are known to play a role in modulating the regulation of plant development under reduced blue light conditions [46]. Their action is independently exerted, but more often the complex regulation of BR signaling involves a cross-talk with other stress responses, such as the ABA [47]. Another plant hormone, cytokinin, was affected by sub-optimal light intensity regardless of the genotype. Cytokinin-mediated developmental regulation, such as shoot growth, leaf senescence, and other photomorphogenic responses, is strongly dependent on light’s characteristics [48]. Our analysis confirmed the light-dependent regulation of cytokinin and derived pathways. Cytokinin synthesis was down accumulated in response to sub-optimal light intensity, and, consequently, the decrease of galactolipids remodeling and porphyrins was observed. Galactolipids synthesis is finely regulated by light, plant hormones, and various stress conditions to maintain the appropriate ratio of monogalactosyldiacylglycerol (MGDG) and digalactosyldiacylglycerol (DGDG) in thylakoid membranes. Here, we observed a galactolipids remodeling with the accumulation of MGDG and a decrease of DGDG when comparing optimal- vs. sub-optimal light intensities, suggesting a putative modulation of DGDG synthases.

Finally, the carbohydrate biosynthetic pathway was also affected by limited light conditions in the red genotype. This result is consistent with the reduced carbon assimilation we observed, which can be explained considering that starch transiently accumulates during the day to be degraded in the darkness. This requires tight regulation of the pathways of starch synthesis and degradation in response to light signals [49].

Taken together, these results indicate that several pathways are modulated as a response to different nutrient solution strengths in Salanova lettuces. However, most of the investigated pathways were cultivar-dependent, with the red-pigmented genotype being more responsive to the treatments compared to the non-pigmented genotype. ‘Red Salanova’ metabolome was characterized by plant stress markers (e.g., ABA, JA), suggesting a possible higher sensitivity compared to green genotype but also its ability to more strongly cope and respond to abiotic factors.

Theknowledge of the diverse response of different genotypes provides insights into plant stress physiology but at the same time suggests possible strategies for the enhancement of phytochemical content of lettuce for biotechnological purposes. Furthermore, the adoption of low input smart agricultural strategies to increase sustainability should measure also metabolic effects in plants in addition to yields and economic aspects. Nonetheless, the occurrence of positive (e.g., the stress-related modulation of functional components) or disadvantageous alterations should be expected when sub-optimal growth conditions are adopted.

In summary, our work indicated that the lettuce metabolomics are easily modifiable and highly plastic and that the two major environmental variations are associated with an extensive cultivar-dependent metabolic alteration. Regardless of the here analyzed lettuce-specific changes, the distinct response of the two cultivars also provides insights on the within-species magnitude of the response to suboptimal conditions that can be expected when analyzing plants that differ phenotypically because of their phytochemical profile. Our results also open the way for the development of strategies to enhance the phytochemical content of lettuce for biotechnological purposes. Finally, our work implies that the effects of low-input sustainable farming systems should be evaluated considering a range of metabolic effects in crops in addition to yield and other socio-economic attributes.

## 4. Materials and Methods

### 4.1. Growth Chamber Conditions, Plant Material, and Treatments

This work was carried out using two lettuce (*Lactuca sativa* L. var. capitata) butterhead cultivars, ‘Green Salanova’ and ‘Red Salanova’ (Rijk Zwaan Italia, Bologna, Italia). Plants were located in a growth chamber equipped with high pressure sodium (HPS) lamps using the Nutrient Film Technique. Experimental set-up conditions were as previously described [26,50]. Briefly, plants grew in with a density of 15.5 plants per square meter, with a 12 h light−12 h dark photoperiod. Air temperature was set at 24–18 °C (light−dark). We used three levels of decreasing macrocation concentrations in the nutrient solution (experiment 1), namely full strength (FS), half strength (HS), and quarter strength (QS), with the FS nutrient solution representing the reference condition. Information on the composition of the nutrient solutions is as previously reported [26]. The macroelements of the FS solution were: 9.0 mM nitrate, 2.0 mM sulfate, 4.0 mM phosphorus, 4.0 mM calcium, 1.0 mM magnesium, and 1 mM ammonium. The factor light had two levels (experiment 2), namely optimal light (OL), with a photosynthetic photon flux density (PPFD) of 420 μmoL m^−2^ s^−1^ and low light (LL), with a PPFD of 210 μmoL m^−2^ s^−1^ [50]. In both experiments, all treatments were replicated three times. Further information on other plant parameters (e.g., leaf area, yield, mineral composition) is presented elsewhere [26,50].

### 4.2. Physiological Parameters

In both experiments, 17 days after transplanting, the following physiological measurements were determined with a leaf gas exchange analyzer (model LCA-4, ADC BioScientific Ltd., Hoddesdon, UK) equipped with a 6.25 cm^2^ broadleaf chamber: net carbon dioxide assimilation rate (A_CO2_), stomatal resistance (r_s_), and transpiration (E). The leaf gas exchange parameters were quantified on 10 red and green pigmented lettuce plants per treatment by choosing one young fully expanded leaf per plant. Intrinsic water use efficiency (WUEi) was obtained as a ratio between net photosynthetic CO_2_ rate and transpiration. The physiological data in both experiments were analyzed by the two-way analysis of variance followed by post hoc analysis (Duncan multiple range; *p* < 0.05) or the independent *t*-test according to the level of fixed factors, namely cultivar (two levels) and NS concentration (three levels) in experiment 1 and cultivar (two levels) and light intensity (two levels) for experiment 2. Statistical analysis was carried out using the SPSS software version 21 (IBM).

### 4.3. Untargeted Metabolomics

For metabolomics analysis, leaves were harvested, immediately frozen in liquid nitrogen, and stored at −80 °C. Freeze dried samples (1.0 g, dry weight) were extracted in 20 mL of 0.1% formic acid in 80% methanol aqueous solution with an Ultra-Turrax (Ika T-25, Staufen, Germany) and centrifuged (12,000 g). Untargeted metabolomics was carried out by using an ultra-high-pressure liquid chromatography coupled to a quadrupole-time-of-flight UHPLC-QTOF mass spectrometer from Agilent (Santa Clara, CA, USA), as reported previously [51]. Briefly, a G6550 iFunnel QTOF mass spectrometer coupled to a 1290 ultra-high-performance liquid chromatograph through an electrospray ionization source (Agilent technologies, Santa Clara, CA, USA) was used. Chromatography was performed using a reverse-phase Agilent pentafluorophenyl (PFP) column (2.0 × 100 mm, 3 µm) (Santa Clara, CA, USA) with a mobile phase of acetonitrile in water (6% to 94%) acidified with formic acid in 33 min with flow rate 200 µL min^−1^. The mass spectrometer was operated in SCAN mode (100–1000 m/z) and positive polarity. Raw spectral data were processed using the targeted “find-by-formula” algorithm using Agilent Profinder B.07 software, (Santa Clara, CA, USA) followed by mass (5 ppm) and retention time (0.05 min) alignment [52]. Compounds were putatively annotated based on the PlantCyc 12.6 database (Plant Metabolic Network; Release: April 2018) by a combination of monoisotopic mass and isotopes ratio and spacing, according to Level 2 with reference to COSMOS Metabolomics Standards Initiative [53]. Compounds annotated in at least 75% of replicates within at least one treatment were retained for subsequent analysis. Quality controls (QCs) were used to obtain a higher degree of confidence in annotation using the MS-DIAL 3.98 (RIKEN Center for Sustainable Resource Science: Metabolome Informatics Research, Yokohama, Japan) to compare the MS/MS spectra to the publicly available MS/MS experimental spectra built in the software (e.g., MoNA) [54].

### 4.4. Chemometric Interpretation of Metabolites

Chemometric interpretation of metabolites was performed using Mass Profiler Professional B.12.06 from Agilent (Santa Clara, CA, USA) as described [51]. Compounds abundance was log2 transformed, normalized at the 75th percentile, and baselined against the median. The unsupervised hierarchical cluster analysis (HCA) was carried out based on fold-change values with the Wards agglomerative algorithm of the Euclidean distances. OPLS-DA supervised analysis was carried out with SIMCA 16 (Umetrics, Sweden) at default parameters. CV-ANOVA (*p* < 0.01) and permutation testing (*n* = 200) were also applied to validate and to exclude overfitting. Goodness-of-fit R2Y and goodness-of-prediction Q2Y were also calculated from the OPLS-DA model. Outliers were investigated according to Hotelling’s T2 (95% and 99% confidence limit for suspect and strong outliers, respectively). Subsequently, a VIP analysis was used to select the most discriminant compounds; such compounds were then subjected to fold-change analysis by comparing the treatments to the optimal conditions. These compounds were analyzed with the Omic Viewer Pathway Tool of PlantCyc (Stanford, CA, USA) to identify pathways affected by the treatments [55]. Venn representation was performed by with Venny 2.1 (https://bioinfogp.cnb.csic.es/tools/venny/).

## 5. Conclusions

The impact of suboptimal nutritional or light inputs was investigated in lettuce within the framework of sustainable agriculture. Photosynthesis-related physiological assessments and metabolomics were used to unravel the biochemical changes underlying the response to limiting growth conditions in butterhead lettuce. Overall, the data indicated markedly different responses between pigmented (red) and non-pigmented (green) lettuce cultivars.

The specific metabolomic signatures we observed involved a broad modulation of metabolism, including secondary metabolites (with nitrogen-containing metabolites and phenylpropanoids explaining the largest differences) as well as phytohormone profiles and the membrane lipids signaling cascade.

## Figures and Tables

**Table 1:** Effect on the strength of the nutrient solution (full strength: FS; half strength: HS; quarter Strength: QS) on net CO_2_ assimilation rate (A_CO2_), stomatal resistance (r_s_) stomatal conductance (E), and intrinsic water use efficiency (WUEi). All data are expressed as mean ± s.e.; *n* = 3.

Source of Variance	A_CO2_		r_s_		E		WUEi	
	(μmol CO_2_ m^−2^ s^−1^)		(m^2^ s^−1^ mol^−1^)		(mol H_2_O m^−2^ s^−1^)		(μmol CO_2_ mol^−1^ H_2_O)	
Cultivar (C)								
‘Green Salanova’	7.86	±0.39 b	4.15	±0.23 b	2.74	±0.06 a	2.89	±0.15 b
‘Red Salanova’	9.88	±0.43 a	5.82	±0.32 a	2.42	±0.06 b	4.14	±0.20 a
*t*-test	***		***		***		***	
Nutrient solution concentration (S)								
FS (EC = 1.50 dS m^−1^)	10.36	±0.46 a	4.69	±0.41	2.70	±0.10	3.96	±0.28 a
HS (EC = 0.75 dS m^−1^)	9.00	±0.42 b	5.10	±0.33	2.53	±0.06	3.61	±0.22 a
QS (EC = 0.50 dS m^−1^)	7.26	±0.52 c	5.16	±0.46	2.51	±0.09	2.97	±0.25 b
	***		ns		ns		**	
C × S								
‘Green Salanova’ × FS	9.29	±0.69	3.68	±0.38	2.92	±0.11	3.23	±0.30
‘Green Salanova’ × HS	7.83	±0.49	4.51	±0.30	2.61	±0.08	3.01	±0.20
‘Green Salanova’’ × QS	6.46	±0.48	4.27	±0.48	2.69	±0.12	2.43	±0.21
‘Red Salanova’ × FS	11.43	±0.30	5.71	±0.54	2.48	±0.12	4.70	±0.31
‘Red Salanova’ × HS	10.17	±0.35	5.70	±0.51	2.44	±0.09	4.22	±0.24
‘Red Salanova’ × QS	8.05	±0.86	6.05	±0.68	2.33	±0.11	3.50	±0.38
	ns		ns		ns		ns	

The symbol “ns” or asterisks (**, ***) indicate a non-significant or significant (*p* ≤ 0.01, and 0.001, respectively) statistical difference. Within a column, different letters (a–c) indicate different statistical groups according to Duncan’s multiple range test (*p* = 0.05). The effect of the cultivar factor was analyzed with a Student’s *t*-test.

**Table 2:** Effect on the light intensity (optimal light: OL; low light: LL) on net CO_2_ assimilation rate (A_CO2_), stomatal resistance (r_s_) stomatal conductance (E), and intrinsic water use efficiency (WUEi). All data are expressed as mean ± s.e.; *n* = 3.

Source of Variance	A_CO2_	r_s_	E	WUEi
	(μmol CO_2_ m^−2^ s^−1^)	(m^2^ s^−1^ mol^−1^)	(mol H_2_O m^−2^ s^−1^)	(μmol CO_2_ mol^−1^ H_2_O)
Cultivar (C)				
‘Green Salanova’	7.53 ± 0.52 b	5.07 ± 0.49	2.54 ± 0.12	2.94 ± 0.13 b
‘Red Salanova’	10.60 ± 0.83 a	5.54 ± 0.76	2.84 ± 0.17	3.67 ± 0.11 a
*t*-test	**	ns	ns	***
Light intensity (L)				
OL (420 μmol m^−2^ s^−1^)	11.61 ± 0.59 a	3.00 ± 0.19 b	3.20 ± 0.11 a	3.63 ± 0.13 a
LL (210 μmol m^−2^ s^−1^)	6.53 ± 0.27 b	7.61 ± 0.27 a	2.19 ± 0.02 b	2.99 ± 0.13 b
*t*-test	***	***	***	***
C × L				
‘Green Salanova’ × OL	9.45 ± 0.30 b	3.34 ± 0.31 c	2.90 ± 0.15 b	3.30 ± 0.16
‘Green Salanova’ × LL	5.62 ± 0.11 d	6.81 ± 0.23 b	2.18 ± 0.05 c	2.59 ± 0.10
‘Red Salanova’ × OL	13.76 ± 0.25 a	2.67 ± 0.17 c	3.49 ± 0.06 a	3.96 ± 0.12
‘Red Salanova’ × LL	7.43 ± 0.23 c	8.41 ± 0.25 a	2.20 ± 0.02 c	3.39 ± 0.12
	***	***	**	ns

The symbol “ns” or asterisks (**, ***) indicate a non-significant or significant (*p* ≤ 0.01, and 0.001, respectively) statistical difference. Cultivar and light intensity factors are compared according to Student’s *t*-test. For factor interactions, within a column, different letters (a, b) indicate significant differences, according to Duncan’s multiple range test (*p* = 0.05).

## Supplementary Materials

Supplementary materials can be found at https://www.mdpi.com/1422-0067/21/17/6381/s1, Figure S1. Unsupervised hierarchical cluster analysis (Euclidean distance; linkage rule: Ward) carried out from ‘Green’ and ‘Red Salanova’ chemical profiles subjected to different concentrations of nutrient solution. Metabolites were obtained by UHPLC-ESI/QTOF-MS untargeted analysis, and their intensities were used to build up the fold-change heatmap here provided. Figure S2. Venn diagram summarizing the discriminant metabolites that resulted from the variable importance in projection (VIP) analysis considering as a factor the response of each cultivar to the variation of nutrient solution concentration. Figure S3. Unsupervised hierarchical cluster analysis (Euclidean distance; linkage rule: Ward) carried out from ‘Green’ and ‘Red Salanova’ chemical under optimal and sub-optimal light conditions. Metabolites were obtained by UHPLC-ESI/QTOF-MS untargeted analysis, and their intensities were used to build up the fold-change heatmap here provided. Figure S4. Venn diagram summarizing the discriminant metabolites that resulted from the VIP analysis considering as a factor the response of each cultivar to the sub-optimal light condition. Table S1. Dataset from untargeted metabolomics of ‘Red’ and ‘Green Salanova’ subjected to different concentrations of nutrient solution. Compounds are presented with individual intensities and with composite mass spectra. Table S2. Discriminant metabolites identified by the VIP analysis in ‘Red Salanova’ subjected to different concentrations of nutrient solution. Compounds were selected as discriminant by possessing a VIP score >1.3 and were uploaded to the PlantCyc pathway Tool software for the subsequent analysis. Table S3. Discriminant metabolites identified by the VIP analysis in ‘Green Salanova’ subjected to different concentrations of nutrient solution. Compounds were selected as discriminant by possessing a VIP score >1.3 and were uploaded to the PlantCyc pathway Tool software for the subsequent analysis. Table S4. Dataset from untargeted metabolomics of the ‘Red’ and ‘Green Salanova’ under optimal and sub-optimal light conditions. Compounds are presented with individual intensities and with composite mass spectra. Table S5. Discriminant metabolites identified by the VIP analysis in ‘Red Salanova’ under optimal and sub-optimal light conditions. Compounds were selected as discriminant by possessing a VIP score > 1.3 and were uploaded to the PlantCyc pathway Tool software for the subsequent analysis. Table S6. Discriminant metabolites identified by the VIP analysis in ‘Green Salanova’ under optimal and sub-optimal light conditions. Compounds were selected as discriminant by possessing a VIP score > 1.3 and were uploaded to the PlantCyc pathway Tool software for the subsequent analysis.

## Abbreviations

HCA	Hierarchical cluster analysis
OPLS-DA	Orthogonal projection to latent structures discriminant analysis
FC	Fold change
VIP	Variable importance in projection
PC	Phosphatidylcholine
PL	Phospholipases
ROS	Reactive oxygen species
MGDG	Monogalactosyldiacylglycerol
DGDG	Digalactosyldiacylglycerol
JA	Jasmonic acids
ABA	Abscisic acid
BR	Brassinosteroids
