# Supplementary figures and images for: The Metabolic Reprogramming Induced by Sub-Optimal Nutritional and Light Inputs in Soilless Cultivated Green and Red Butterhead Lettuce
Source: Int J Mol Sci. 2020 Sep 2;21(17):6381. doi: 10.3390/ijms21176381 (PMC7503926; doi:10.3390/ijms21176381)

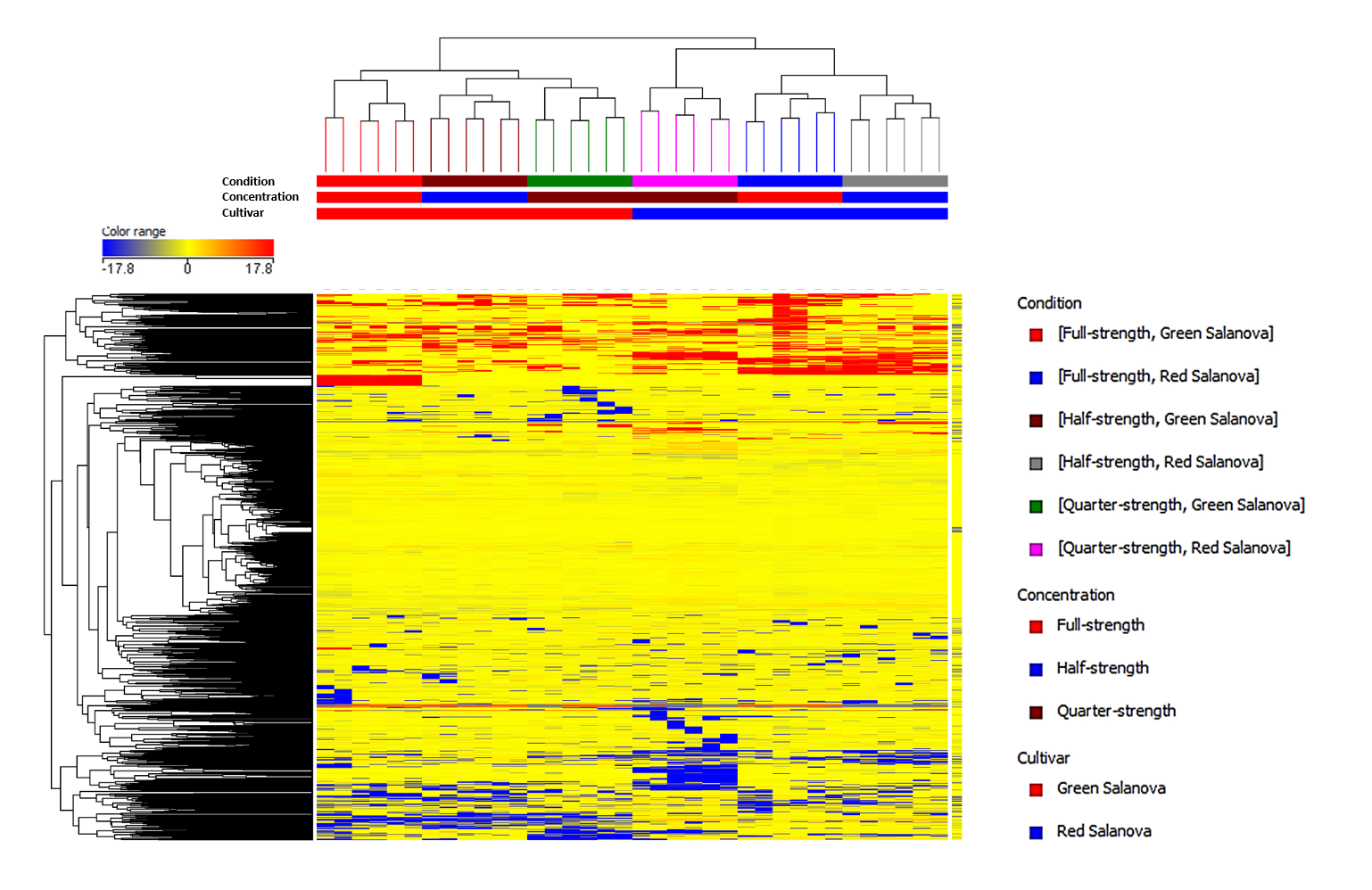

Supplement: Supplementary file 1 [file ijms-21-06381-s001.zip › Supplementary Submission/Supplementary Figure 1.tif]

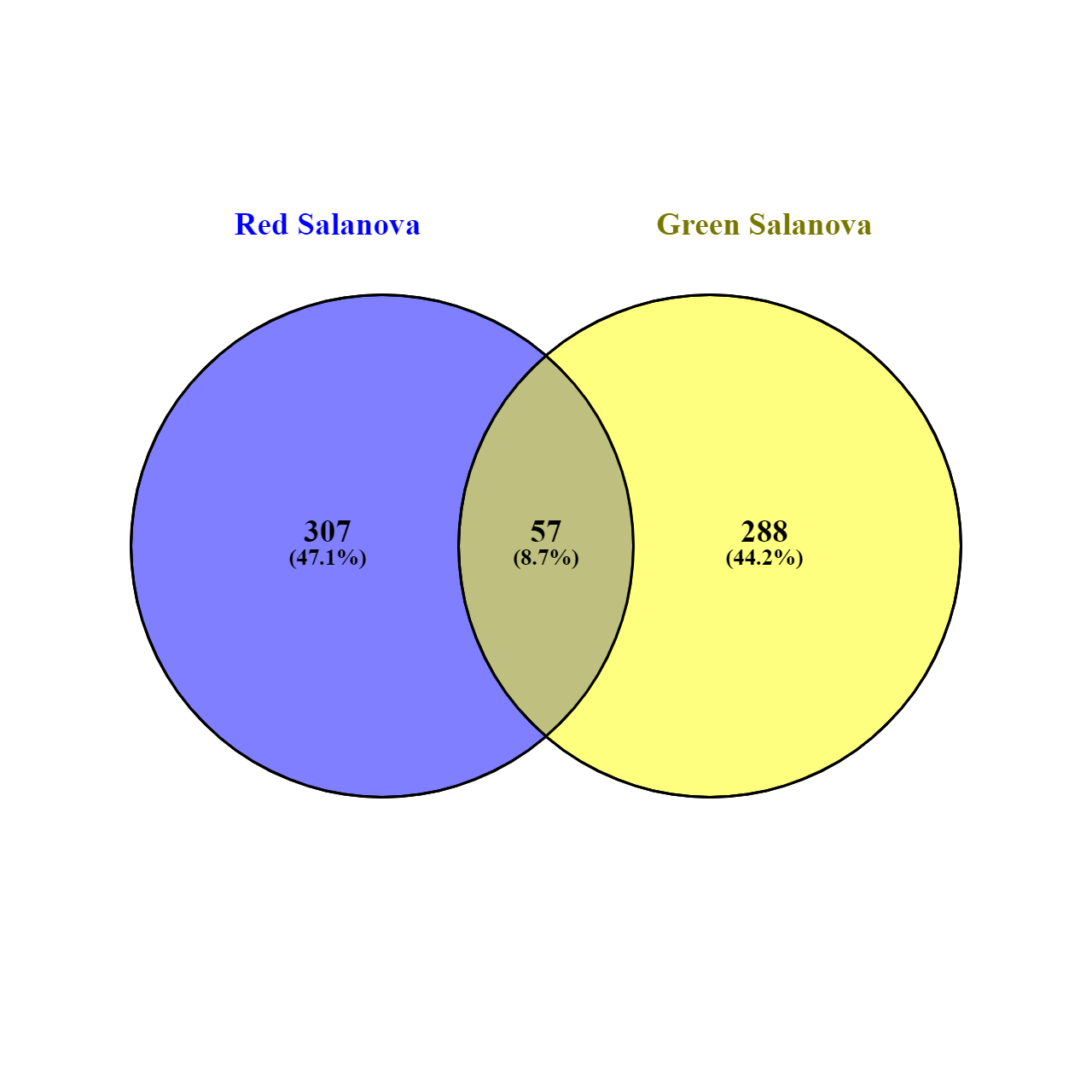

Supplement: Supplementary file 1 [file ijms-21-06381-s001.zip › Supplementary Submission/Supplementary Figure 2.png]

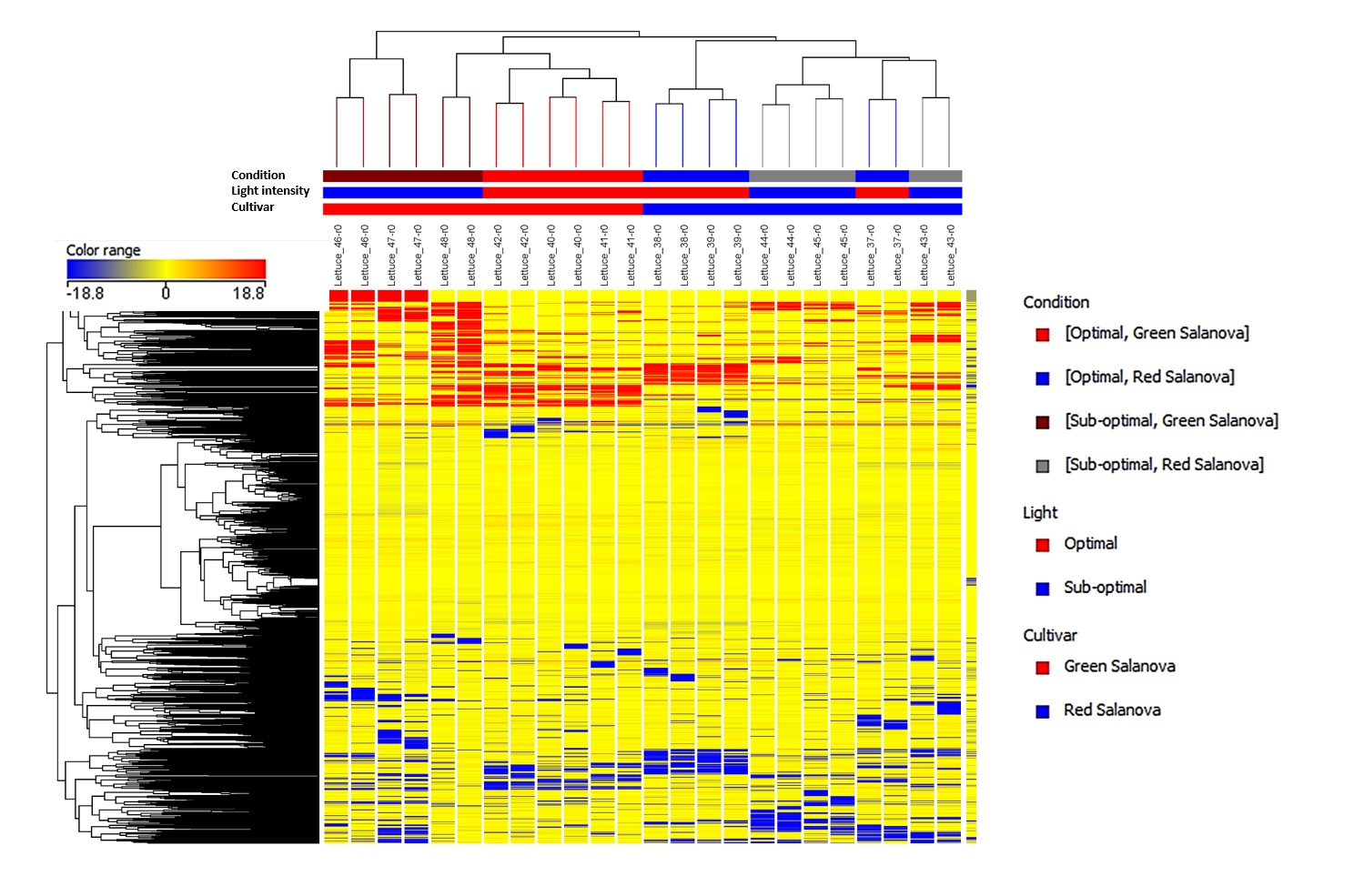

Supplement: Supplementary file 1 [file ijms-21-06381-s001.zip › Supplementary Submission/Supplementary Figure 3.tif]

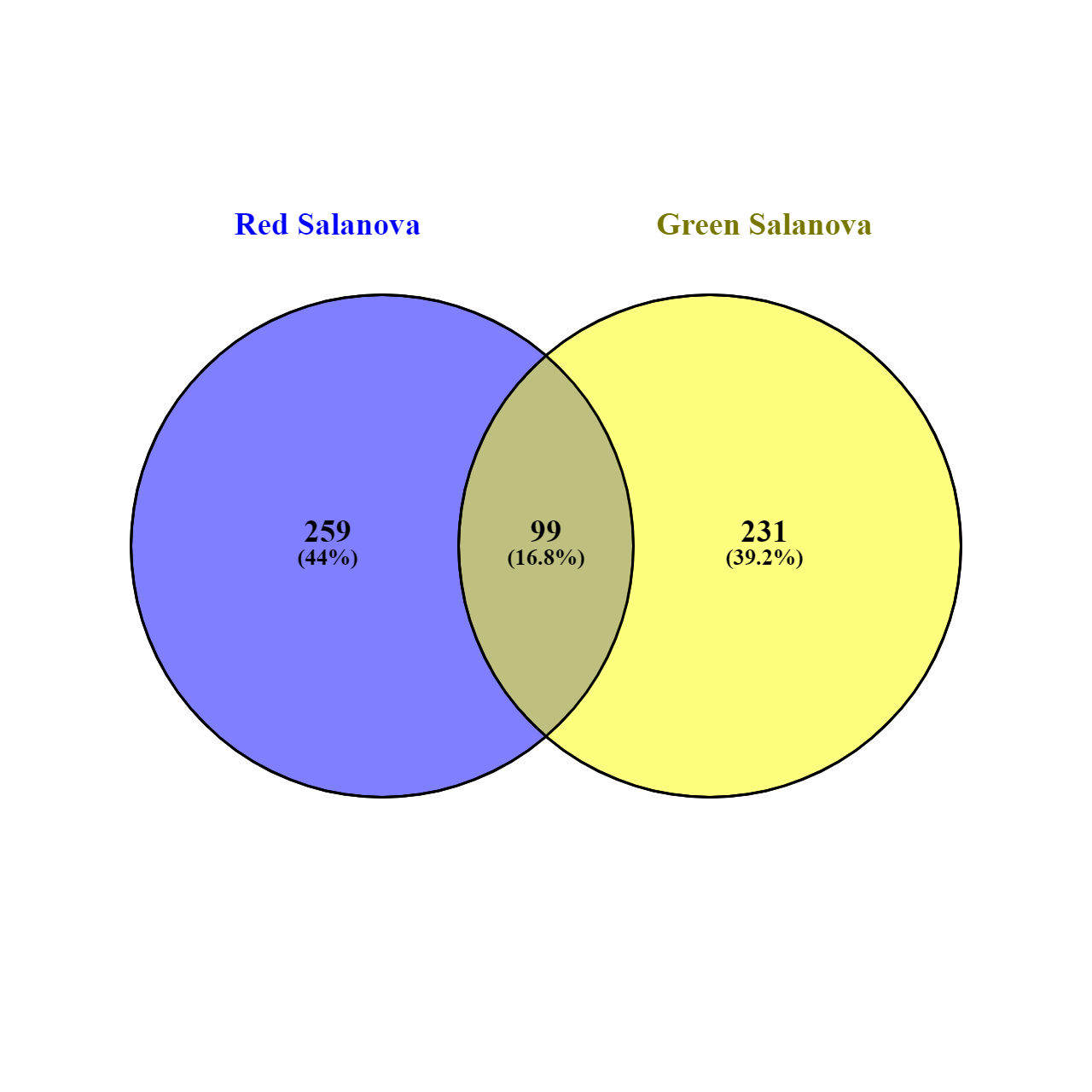

Supplement: Supplementary file 1 [file ijms-21-06381-s001.zip › Supplementary Submission/Supplementary Figure 4.png]
